# Impact of flipped classroom-based simulation of CPR on nursing students’ self-confidence, satisfaction, knowledge and skill: a quasi-experimental study

**DOI:** 10.1186/s12909-025-07525-9

**Published:** 2025-07-01

**Authors:** Shokoufeh Nasiri, Reza Hosseinabadi, Yaser Mokhayeri, Shourangiz Beiranvand

**Affiliations:** 1 Medical-Surgical Nursing, Student Research Committee, Lorestan University of Medical Sciences, Khorramabad, Iran; 2 Nursing Department, Social Determinants of Health Research Center, Pardis Kamalvand Lorestan University of Medical Sciences, PO Box: 6814993165, Khorramabad, Iran; 3 Cardiovascular Research Center, Shahid Rahimi Hospital, Lorestan University of Medical Sciences, Khorramabad, Iran

**Keywords:** Cardio pulmonary resuscitation, Nursing students, Self confidence, Nursing education

## Abstract

**Background:**

Nursing students frequently exhibit inadequate knowledge and performance and low self-confidence and satisfaction levels regarding cardiopulmonary resuscitation (CPR) training methods. Innovative educational approaches allow millennial students to engage in self-directed learning styles. Hence, this study aims the impact of Flipped Classroom-Based Simulation (FCBS) on self-confidence and satisfaction, knowledge, and skill of CPR in third-year nursing students.

**Methods:**

This quasi-experimental study, conducted in 2023, involved 75 third-year nursing students from Lorestan University of Medical Sciences, Iran. The participants were randomly assigned to either the intervention group (*N* = 38) and control group (*N* = 37). the intervention group received training CPR knowledge and skills with the approach of FCBS. The control group received training CPR knowledge and skills using the simulation based education (SBE) method. The duration of training for each group was three hours per week. The total intervention time was 15 h. Data collection included a demographic information inventory; a scale for evaluating satisfaction and self-confidence in learning; a questionnaires assessing CPR knowledge and a checklist for evaluating CPR skills. Both groups completed questionnaires before and 2 weeks after the educational intervention. Data were analyzed using descriptive statistics, independent t-tests, paired t-tests, and ANCOVA with Stata-17 software.

**Results:**

The results showed that the mean total score for self-confidence and satisfaction, CPR knowledge and skills in nursing students was significantly higher in the intervention group (*P* = 0.001). Intergroup analysis using ANCOVA showed a statistically significant difference in the changes in the mean total score knowledge of CPR and its subscales, except for the subscale concerning the principles of artificial ventilation and airway management, with (P= 0.26).

**Conclusion:**

This innovative educational approach can promote active learning and greater student engagement in CPR training. It is recommended that educators adopt this method in CPR education to improve student knowledge, skills, and satisfaction.

## Background

Cardiopulmonary arrest is a critical healthcare burden with profound socio-economic consequences. It accounts for 15–20% of all natural adult deaths in the United States and Western Europe [1]. Nurses and nursing students must be competent to initiate and perform cardiopulmonary resuscitation (CPR) effectively in their nursing profession [2]. According to available studies, the CPR skills among nursing students are not at a desirable level. studies in Iran showed that nursing students scored only 59.05% in knowledge and 70.12% in skills related to CPR. Moreover, their skill scores fluctuated over time, with declines observed in some cases [3, 4]. There are gaps in the knowledge and practical skills of professionals in performing high-quality CPR, which can impact the outcomes and survival chances of patients after CPR arrest [5].

Currently, there is an emphasis on utilizing innovative nursing teaching approaches that enhance the quality of care, improve nursing competencies, and bridge the gap between theory and practice [6]. Therefore, to enhance students’ knowledge and proficiency in CPR, it is necessary to consider innovative educational methods that promote better, deeper, and more lasting learning [7]. The flipped classroom is one of the innovative teaching approaches for nursing students, with the potential to transform the teaching-learning process in nursing education [8]. In this method, the educator plays an interactive role in the teaching-learning process, acting as a facilitator and guide, providing feedback to learners, and fostering a collaborative and participatory educational environment [9]. This approach is student-centered, allowing students to utilize various technologies to understand educational content before attending class and then apply and practice this information under the educator’s supervision [6].

Simulation Based Education (SBE) is a standard method for providing CPR learning. It has a disadvantage in which the students are relatively passive, only the instructors actively involved in the learning process. However, the researchers founded that the traditional simulation teaching methods have not fully mobilized the active participation of nursing students, and the theory and practice are not closely combined [10]. Hassan and Elsaman found that in CPR skills training to nursing students, the mean score of the CPR knowledge and skill and satisfaction in the Flipped Classroom-Based Simulation (FCBS) group was significantly higher than traditional simulation group [11]. The International Nursing Association for Clinical Simulation and Learning (INACSL) emphasizes the importance of preparatory activities before simulation to achieve participants the intended simulation objectives. Health care students often participate in simulation sessions without proper preparation, which hinders the achievement of the desired learning outcomes [12]. Additionally, SBE can increase stress due to tension and anxiety from real-life scenarios, fear of making mistakes, limitations in problem-solving processes, and reduce students’ self- confidence and learning satisfaction [11]. A review of the literature suggests integrating simulation with the flipped learning model [13]. However, the combination of these two methods has not yet been quantitatively tested in a well-designed study. SBE requires aneducational method that enhances its impact on students learning [14].

Therefore, a teaching and learning method, as an innovative educational approach, is essential for overcoming the limitations and challenges of SBE [15]. FCBS is a specific type of flipped learning that emphasizes clinical skills and to the essential knowledge foundational required for performing these skills. Considering that, enhancing nursing students’ skills is the cornerstone of nursing education, the proposed teaching method is effective in raising the high level of competence expected in undergraduate nursing students’ skills [16].This teaching-learning method consists of two components: learning practical topics through video outside the classroom, followed by experiential learning through various types of simulation or face-to-face environments, the learners engage in experimental learning through simulation of various sorts. The debriefing phase of FCBS, learners can react and reflect on their practice, Thus, learners identify how they perceive, process, and learn the information they provided and experience [17]. Reported benefits of the flipped classroom include: increased learner motivation and engagement, improved student learning performance, ownership and monitoring of their own learning, and increased interaction among students [18]. Additionally, this learning style allows them to practice the concepts learned prior to class in clinical settings, facilitating the transition from theory to clinical practice [19].

The flipped classroom is based on John Dewey’s active learning theory. According to Dewey, active learning involves active engagement in the learning process, encouraging critical thinking, problem solving, and experimentation. Active learning theory is an educational approach that emphasizes the active involvement of the learner in acquiring knowledge and skills. The theory is based on the idea that individuals learn best when they are actively involved in their learning, rather than being passive receivers of information [20]. Student’s engagement and active participation in learning process results in better achievement and increase in student’s self-confidence and satisfaction. Therefore, it is crucial to take nursing students confidence and satisfaction into account during nursing education. In the flipped classroom approach, students’ active role in their own development and skill enhancement during the learning process leads to increased self-confidence [21]. self-confidence is defined as students’ belief in their knowledge and practical skills in providing nursing care. Nursing students also need self-confidence to face complex clinical situations and provide safe, accurate, and high-quality care to patients. Keleekai et al. (2016) found that implementing a blended program, including online training and simulation, significantly improved nurses’ knowledge, self-confidence, and skills in peripheral intravenous catheter placement [22]. In FCBS, satisfaction can be considered an important component not only for success in achieving the desired goal, but especially for the positive reinforcement of self-confidence and in the experiences that will build a future professional profile [23]. The findings of the studies demonstrated that the flipped classroom approach effectively improved academic achievement, knowledge and skills, self-confidence and perception of nursing students compared to conventional teaching methods, moreover, after training, high levels of satisfaction and self-confidence were reported [24, 25].

In health-related sciences, despite the growing trend in the use of FCBS training, there are few studies on the implementation of this educational approach in nursing programs [26]. The application of flipped classroom in nursing education is still in its early stages [27]. Most studies have been international in nature, focusing on performance and academic achievement, primarily used for theoretical courses, and have been less frequently applied in practical courses [28]. As previously noted, the knowledge and skills of nursing students, who are primarily taught through traditional methods, remain inadequate, presenting challenges for nursing instructors. This may be attributed to the teaching methods currently employed. Consequently, the adoption of modern educational approaches that encourage active student engagement in the learning process could potentially lead to more effective outcomes in teaching CPR to nursing students. Therefore, we aimed to the impact of a FCBS approach of CPR on self-confidence, satisfaction, knowledge and skill of third-year nursing students.

## Methods

### Design and setting

This pre-post-test quasi-experimental study was undertaken at the School of Nursing and Midwifery, Lorestan University of Medical Sciences, between September to December 2023.

### Participants and sampling

The study sample comprised 75 third year nursing students enrolled in semesters the sixth and fifth of Khorramabad Nursing School (West Iran) were invited to participate in the study. The blocked randomization method was used. Randomized codes numbers were generated by a research assistant by Stata software in four blocks. A code was assigned to each student, subsequently, then randomly assigned to either the intervention (*N* = 38) and the control (*N* = 37) groups. The research assistant only assisted in recruiting participants and was not involved in data collection and analysis. The intervention group received FCBS training, an approach that the students had not previously experienced, while the control group received SBE.

To be eligible for inclusion in the study included the willingness to participate in the study, lack of the previous familiarity with the flipped classroom, taking the emergency care nursing course for the first time and access to a computer or mobile phone with internet to download PowerPoint files and short videos through Lorestan Medical Science (L.M.S). The content-sharing platform was the L.M.S. of Iran Virtual University of Medical Sciences. Exclusion criteria included absence from training sessions more than once, participation in CPR skills or promotion of emergency nursing courses during the intervention and failing to complete the questionnaire.

## Tools and measurements

### Demographic information form

The demographic information form included age, gender, academic semester, dormitory status, interest in nursing, and grade-point average (GPA) of the previous semester. In terms of face validity, ten Khorramabad Nursing and Midwifery Faculty members reviewed and approved this form.

### Satisfaction and self- confidence scale in learning

This instrument consists of 13 items developed by the National League for Nursing and includes two subscales: one for measuring student satisfaction (five items) and the other for assessing self- confidence in learning through simulation (eight items). Scores range from 5 to 25 for the satisfaction subscale and from 8 to 40 for the self- confidence subscale. Each item was scored on a 5-point Likert scale ranging from 1(strongly disagree) to 5 (strongly agree). Higher scores indicating greater satisfaction and self-confidence [29]. In Iran, original version of the tool was translated from English to Farsi simultaneously by two experienced translators fluent in English. reliability coefficients of the Persian version of the satisfaction and self-confidence scale in learning with Cronbach’s alpha was obtained 0.83 and 0.84, respectively [30].

### The questionnaire of CPR knowledge: basic life support (BLS) and advanced life support (ALS) and knowledge in the use of defibrillator

The questionnaire of CPR knowledge consisted of two parts. The first part assessed the levels of BLS and ALS knowledge of CPR using a questionnaire developed by Pourmirza Kalhori et al. This questionnaire contained 40 items regarding CPR knowledge in four domains: (1) Main rules of CPR initiation and termination, (2) The principles of establishing artificial ventilation and management of airway, (3) the principles of external chest compressions and (4) advanced resuscitation principles [31]. Five experts in intensive care and emergency nursing at the nursing faculty of Lorestan University of Medical Sciences, each with over 10 years of experience in emergency nursing, evaluated the instrument for clarity, relevance, comprehensiveness, and appropriateness. Following the incorporation of specialists’ feedback. It was increased to 44 items based on the American Heart Association Adult CPR Guidelines Association [32, 33]. The reliability of this tool was determined using Cronbach’s alpha, which was found to be 0.68. Responses to the questionnaire were in a yes/no format, with a correct answer scoring 1 point and an incorrect answer receiving no points. Total scores ranged from 0 to 44, higher scores indicating greater knowledge.

The second part was the measurement of knowledge in the use of defibrillator, which was used by Dehghani et al.‘s questionnaire. The content validity index (CVI) and content validity ratio(CVR) coefficient were obtained as 0.8 and 0.9, respectively, which indicated a high level of experts’ agreement [34]. The reliability of this instrument using Cronbach’s alpha in this study was 0.80. Scoring was structured such that a correct option received 1 point, while all other options received 0 points.

### The checklist of CPR skills: BLS and ALS skills and skill of working with defibrillator

The checklist of CPR skills consisted of two part. The first part included a checklist for evaluating BLS and ALS skills of CPR, which was derived from the observational checklist by Maden and the 2020 guidelines of the American Heart Association, as utilized in the study by Tari Nejad et al. [35]. The CVR for all questionnaire items was calculated to be greater than 0.79, and the internal consistency reliability (Cronbach’s alpha) for the BLS and ALS skills checklist of CPR was obtained as 0.97. Each checklist included items related to both basic and advanced CPR skills, measured using a 3-point Likert scale (0 = not performed, 1 = partially performed, 2 = fully performed) to assess skill accuracy. The cumulative score for each section is determined by adding together the individual item scores.

The second part included a checklist of the skill of working with defibrillator. This checklist included 15 items about the skills of working with the defibrillator, using a 5-point Likert scale (with scores ranging from 1 to 5). Total scores ranged from 15 to 75. The content validity of this tool was established, and its reliability was determined using Cronbach’s alpha, which was found to be 0.76, as reported by Dehghani et al. [34]. In this study, the CVR for all checklist items was calculated to be greater than 0.75, and the CVI was found to be greater than 0.79.

### Emergency care nursing course in Iran

CPR is part of the emergency care nursing course, which consists of half a practical and 0.25 theoretical credits. Routinely, practical CPR training is conducted in 5 sessions with a SBE approach. The educational content for the intervention and control groups is shown in Table 1. The educational materials and videos were developed based on the adult basic and advanced CPR guidelines provided by the American Heart Association [31, 32]. The videos comprised verbal explanations and stepwise demonstrations. Both groups were taught by the second author (Ph.D. of nursing) who had 20 years of experience teaching emergency care nursing courses and conducting CPR workshops for nursing students, and the first author (master’s student in medical-surgical nursing) as co teaching who had five years of clinical experience in the cardiac emergency department.

**Table 1:** Titles of modules for CPR training in two group

Modals	Intervention group	Control group
Session1	Basic Life Support	Basic Life Support
Session2	The skill of working with a defibrillator	The skill of working with a defibrillator
Session3	Intra tracheal Intubation	Intra tracheal Intubation
Session4	Training of CPR Drugs monitoring	Training of CPR Drugs monitoring
Session5	Advance Life Support	Advance Life Support

## Intervention

### Teaching methods in the FCBS

**Fig. 1:**
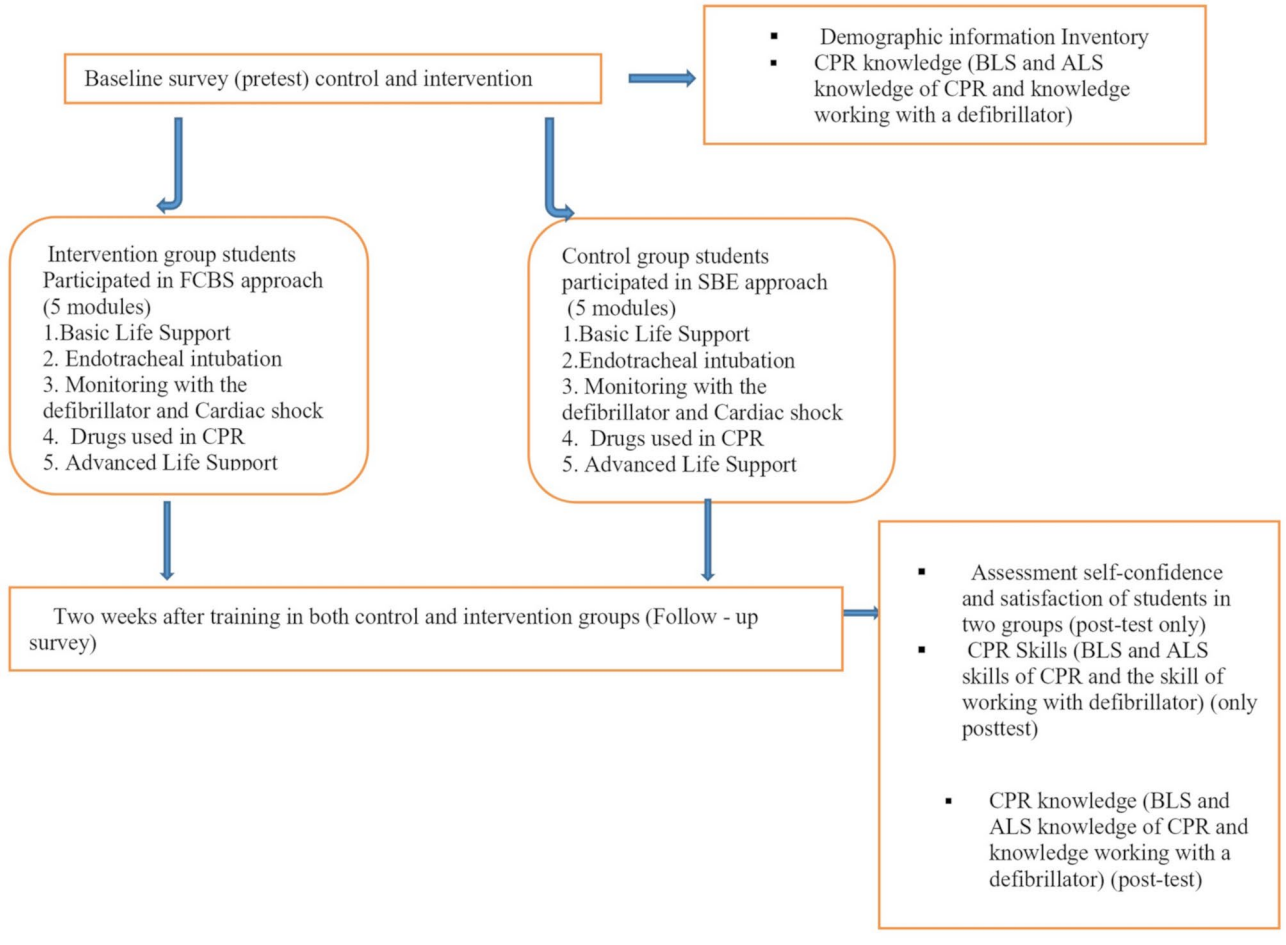
Flipped classroom based simulation and simulation based education teaching procedures

The intervention group received FCBS training. A one-hour orientation session was held for the students of the intervention group one week before the intervention. They received CPR knowledge and skill training for 5 weeks during the second half of the academic semester. It was run for three hours per week (90 min of teaching and 90 min of practice). The total intervention time was 15 h. The procedures for the flipped classroom learning approach and SBE method are shown in Fig. 1.

The video and text content was pre-designed by the educators. One week before the presentation of each psychomotor skill, its educational content was provided to the participants in the form of PowerPoint files and short videos through L.M.S. During the flipped classroom sessions, students were divided into two groups. Classroom seating for each group was arranged in a circular format. The content was delivered equally to both groups based on the lesson plan by the educator and co teaching. Initially, in each group, before starting each session, the educator provided a brief introduction for 10 min about the general and specific goals of the lesson. Then, students practiced CPR knowledge and skills using low-fidelity mannequins in the skills lab with mutual participation for 90 min, explaining as they practiced. The educator primarily listened, answered their questions when necessary, and clarified concepts. The instructor’s role was to provide feedback during practice. After a 20-minute break, the first group practiced the CPR skills using a low-fidelity mannequin for an additional 90 min with the co teaching to reinforce learning with feedback. Students discussed the key points related to the videos and were asked to take notes on the important aspects of the procedures (from their perspective) and reflect on classroom events. This process was repeated in the same manner for the second group.

### Teaching methods in the control group

The control group received CPR skills training using the SBE method for 5 weeks during the first half of the academic semester. It was run for three hours per week (90 min of teaching and 90 min of practice). They were divided into two groups. The training was mainly delivered through lectures accompanied by practical demonstrations on low-fidelity mannequins. If students had questions, they were asked during the training, and feedback was provided by the educator. Thus, the educator played an active role while the students took a passive role. After a 20-minute break and the end of the class, the students practiced CPR again for another 90 min using low-fidelity mannequins with the co teaching assistant to reinforce learning. This procedure was repeated for the second group.

### Data collection methods

After obtaining study approval from the ethics committee of Lorestan University of Medical Sciences, students who provided written informed consent to participate in this study were selected. Prior to the intervention, both groups completed a demographic information form, they also completed the CPR knowledge questionnaire twice: once before the intervention and again 2 week later (pre and posttest). After the intervention, BLS and ALS skills of CPR were assessed through Objective Structured Clinical Examination (OSCE) by assessors. The evaluation took place in the Faculty of Nursing and Midwifery simulation lab, utilizing pre-prepared mannequin’s. It consists three station (Ventilation, chest compressions, and prescription of medication and shock using a defibrillator) lasting 15 min. The evaluators observed the learners’ skills using the checklist for BLS and ALS, skills of CPR during the OSCE. Feedback based on objective findings was then provided to the learners. Both the intervention and control groups were requested to complete self-confidence and student’s satisfaction questionnaire 2 weeks after intervention at the designated location within the nursing (only posttest).

### Data analysis

Descriptive statistics, including mean (x), standard deviation (SD), frequency, and percentage were used to describe the data. The Kolmogorov-Smirnov test was used to ensure the normality of data distribution. A paired t-test was used to examine the changes in mean scores of CPR skills and knowledge regarding the defibrillator before and after the intervention in each of the study groups. Independent t-tests were applied to determine the difference in skill, self-confidence, and satisfaction scores between the two groups after the intervention. Additionally, Analysis of Covariance (ANCOVA) was used to compare the mean scores of quantitative variables in the post-phase between the two groups while adjusting for baseline effects. The Chi-square test was used to examine the relationship between qualitative variables. All analyses were conducted using Stata 17 software, and the significance level for all tests was set at 0.05.

## Results

The findings indicated that there were no statistically significant differences between the two groups in terms of all demographic, age (*P* = 0.15), gender (*P* = 0.9), academic progress (*P* = 0.86), interest in nursing (*P* = 0.73), semester (*P* = 0.12), GPA (*P* = 0.86) and living situation (*P* = 0.74), suggesting that the two groups were homogeneous (Table 2). An independent t-tests was used to evaluate the between group difference in CPR skills and self-confidence and satisfaction of students after the intervention. The results showed that the mean total score for self-confidence and satisfaction of students was significantly higher in the intervention group (53.9 vs. 45.7, *P* = 0.001). Also, the mean scores of the intervention group in the sub-scales of BLS skills of CPR (33.92 vs. 30.18; *P* = 0.001), ALS skills of CPR (31.94 vs. 27.4; *P* = 0.001), and skills for working the defibrillator (58.8 vs. 34.75; *P* = 0.001). The mean scores in the intervention group were higher than those in the control group. Statistical analysis showed a significant difference between the mean scores of the two groups after the intervention (Table 3).

**Table 2:** Demographics of the participants (*N* = 75)

Characteristic	Total *n*= (75)		Intervention *n* (38)	Control *n*(37)	χ2	*P* value
Gender	Male	40(53.4)	20(52.7)	20(45.9)	0.01	0.9
	Female	35(46.6)	18(47.3)	17(54.1)		
Interest in nursing	Yes	56(74.6)	29(76.3)	27(73 )	0.11	0.73
	No	19(25.4)	9(23.7)	10(27)		
Living status	Dormitory	29(38.6)	14(36.8)	15(40.5)	0.10	0.74
	With family	46(61.4)	24(63.2)	22(59.5)		
Academic term	Five	31(41.3)	19(50)	12(32.4)	2.38	0.12
	Six	44(58.7))	19(50)	25(67.5)		
Age	22.78 (2.10)		23.13(2.75)	22.43(1.01)	-1.45	0.15
Grade-Point Average (GPA)	16.98 (0.9)		16.96(0.78)	16.92(1.02)	-0.17	0.86

**Table 3:** Comparison of satisfaction and self-confidence and cardiopulmonary resuscitation skills in two control and intervention groups (post-test)

Variables			Intervention Group Mean ± SD	Control Group Mean ± SD	Mean difference(SD)	Effect size	t^a^	*P*	Confidence interval 95%
Satisfaction		Post	21.39(2.16)	17.4(5.87)	-3.89(1.01)	0.4	-3.92	0.001	-6.01, -1.96
Self-confidence		Post	32.5(3.59)	28.37(7.22)	-4.12(1.31)	0.34	-3.14	0.002	-6.73, -1.50
Total			53.9(5)	45.7(11.9)	-8.11(2.1)	0.4	-3.84	0.001	-12.31, -3.91
CPR skills	ALS	Post	31.94 (2.25)	27.4( 2.79)	-4.54(0.58)	0.6	-7.76	0.001	-5.7, -3.37
	BLS	Post	33.92(2.32)	30.18(3.21)	-3.73(0.64)	0.55	-5.77	0.001	-5.02, -2.44
	The skill of working with a defibrillator	Post	58.8(9.37)	34.75(14.5)	-24.11(2.81)	0.7	-8.55	0.001	-29.72, -18.49

t^a^= (Independent Samples t-test)

A paired t-test was used to evaluate the within group difference in BLS and ALS knowledge of CPR of and its subscales and defibrillator knowledge before and after the intervention. In the intervention group, the results showed that the mean score on defibrillator knowledge varied from 4.63 in pretest to 8.05 in posttest. In the control group, the mean score on the defibrillator knowledge varied from 3.51 in pretest to 5.75 in posttest, which had a significant increase in two groups. The mean total score on the BLS and ALS knowledge of CPR varied from 31.10 in pretest to 36.71 in posttest. In the control group, the mean total score on the BLS and ALS knowledge of CPR varied from 27.75 in pretest to 32.16 in posttest, which had a significant increase in two groups. Also, in terms of subscales of the BLS and ALS knowledge of CPR, in the intervention group compared to the control group, the score of main rules of CPR initiation and termination principles (6.28 vs. 5.13), the principles of establishing artificial ventilation and management of airway (8.89 vs. 8.51), the principles of external chest compressions (10.84 vs. 10) and advanced resuscitation principles (10.68 vs. 8.51) after the intervention had an increase. The results were significant about the subscales of BLS and ALS knowledge of CPR except for the subscale of main rules for starting and ending CPR in the control group (Table 4).

**Table 4:** Comparison of basic and advanced CPR knowledge and defibrillator knowledge in two groups before and after the intervention

Variables			Intervention Group Mean ± SD		^b^ t	Mean difference(SD)	*P*	conf. interval 95%	Control Group Mean ± SD	Mean difference(SD)	t^b^	*P*	conf. interval 95%
CPR knowledge	Knowledge of defibrillator		Pre	4.63(2.37)	-8.78	-3.4	0.001	-4.2,-2.6	3.51(1.67)	-2.24	-5.80	0.001	-3,-1.4
			Post	8.05(1.27)					5.75(2.25)				
	Knowledge of BLS and ALS CPR	The main rules of CPR	Pre	5.1(1.26)	-6.05	-1.1	0.001	-1,5, − 0.78	4.67(1.59)	− 0.45	-1.45	0.15	-1.1,0.18
			Post	6.28(0.83)					5.13(1.08)				
		Management of airway	Pre	7.78(1.64)	-3.38	-1.1	0.001	-1.7, − 0.44	7.48(1.60)	-1.02	-3.32	0.002	-1.6,-0.4
			Post	8.89(1.29)					8.51(1.26)				
		Chest compressions	Pre	8.97(1.73)	-7.8	-1.8	0.001	-2.3, -1.3	8.35(1.61)	-1.6	-4.2	0.001	-2.44,-0.85
			Post	10.84(1.02)					10(1.8)				
		Advanced CPR	Pre	9.23(1.82)	-3.94	-1.44	0.001	-2.19, − 0.7	7.24(1.75)	-1.2	-3.2	0.001	-2,-0.46
			Post	10.68(1.56)					8.51(1.52)				
		Total	Pre	31.10(3.74)	-7.69	-5.6	0.001	-7, -4.1	27.75(3.48)	-4.4	-5.74	0.001	-5.9,-2.8
			Post	36.71( 3.04)					32.16(3.57)				

t^b^= Paired t test

After adjusting the effect of the baseline scores, BLS and ALS knowledge score of CPR and defibrillator knowledge score, between-group ANCOVA analysis revealed a statistically significant difference between the changes in the mean score defibrillator knowledge and total score of BLS and ALS knowledge of CPR and its subscales (The main rules of starting and ending CPR, external chest massage and advanced revival principles) *p* < 0.001. However, no significant difference was observed the changes in the mean score in the subscales of Airway management and artificial ventilation (*p* = 0.26) (Table 5). The intervention group compared to the control group, a significant improvement in was observed.

**Table 5:** Comparison of defibrillator knowledge and basic and advanced CPR knowledge and scores of subscales between two groups at the post-intervention point using ANOVA

Variables			Intervention Group (*N* = 38) Mean ± SD	Control Group (*N* = 37) Mean ± SD	F	*P* value
CPR knowledge	Knowledge of defibrillator		8.05(1.27)	5.75(2.25)	18.22	0.001
	Knowledge of BLS and ALS CPR	The Main rules of CPR initiation and termination	6.28(0.83)	5.13(1.08)	14.52	0.001
		The principles of establishing artificial ventilation and management of airway	8.89(1.29)	8.51(1.26)	1.35	0.26
		The principles of chest compressions massage	10.84(1.02)	10(1.8)	5	0.009
		Advanced resuscitation principles	10.68(1.56)	8.51(1.52)	18.36	0.001
		Total	36.71( 3.04)	32.16(3.57)	18.29	0.001

## Discussion

A short-term intervention with three components was implemented in the present study. This study aimed to investigate the effects of FCBS training on self-confidence and satisfaction and CPR knowledge and skill among third-year nursing students. The study results showed that students’ confidence self-regarding CPR skills significantly increased after participating in the simulation experience with flipped classroom learning. These findings were consistent with the results of Wilson and Hobbs (2022). They founded that the intervention of FCBS effectively strengthened the satisfaction and self- confidence among nursing students regarding the practice of clinical skills [36]. Studies have shown that nursing students with high self- confidence demonstrate better professional clinical practice than those with low self- confidence and can provide superior nursing care to patients [37]. Students in group FCBS had more knowledge and skills than the traditional group. These findings were consistent with the results of Muhibbuddin et al., (2020) [38]. This might be due to students in the FCBS group freely discuss the details of the materials, videos and abilities with their classmates. Deepen your understanding and increase your level of knowledge and skills, leading to confidence and satisfaction.

Furthermore, the results of this study revealed that FCBS training could increase satisfaction in learning compared to those who received SBE training. These findings were consistent with the study’s results of by Gu and Sak (2020) and Chang et al. (2019). They found that FCBS is the most effective teaching and learning method for clinical skills training and advanced cardiac life support. It can lead to improved performance, self-awareness, motivation and satisfaction in learning nursing students [39, 40]. This educational approach causes independence, responsibility and team interaction of students in classroom. It provides opportunities to apply their previous learning in the classroom, which increases the intrinsic satisfaction of learners.

The results of this study revealed that the mean total score of CPR skills in students who underwent FCBS training was higher than that of those who received SBE training. FCBS training allows students to review pre-recorded videos prior to the practical class. It enables them to engage deeply with clinical education while practicing with their peers. This educational approach can facilitate students’ participation in discussions on knowledge and clinical skills [11]. Incorporating background knowledge through flipped learning and procedural skills through simulation can enhance students’ preparation for real clinical situations. A systematic review conducted by Pangandaman et al. (2024) showed that flipped classroom significantly enhance nursing students’ clinical skills, particularly in CPR, urinary catheterization, and safe medication administration [41]. However, a review of the literature reported different results comparing the effects of FCBS and SBE teaching on students’ academic achievement and performance. For instance, Hassan and Elsaman (2023) and Gu and Sak (2020) reported that FCBS training led to more effective improvements in nursing students’ performance compared to the SBE approach. Conversely, current results are somewhat inconsistent with other studies [11, 39]. Hasanvand et al. (2023) and Beom et al. (2018) found no significant differences in student performance regarding the learning of nursing principles and skills and advanced cardiac life support between the FCBS and SBE methods [42, 43]. These differences in the results can be attributed to the various processes, materials and environments utilized in the implementation of the flipped learning model, in addition, the educators who manage this process plan it in different ways and perform various classroom activities. These differences in activities may have produced mixed results.

The present study showed that students who received FCBS training scored higher in the subscales the principles of external chest compressions, main rules of CPR initiation and termination principles and advanced resuscitation principles except for the principles of establishing artificial ventilation and management of airway sub-scale. This can be attributed to the instructor’s emphasis on ensuring that students in both groups practice enough in the same session. Since ventilation is a practical procedure, this training may have been effective enough, therefore no significant change was observed between the two groups. Instructors should be aware of the complexity of this skill and should focus on its correct performance during training following the online learning process. The study by Van Ramdonk et al. (2017) also identified difficulties in learning, ventilation skills, indicating that simulation can effectively enhance skills, provided that the simulation video focuses on the step-by-step process [44].

### Limitations

There are several limitations to our study. The results are based on simulations on mannequins, so it is not clear how students would react in a real situation where they would have to act. Therefore, it is recommended that future research includes observations of nursing students at the patient’s bedside during CPR to strengthen this innovative learning method further. The study sample being limited to a specific group of nursing students from Lorestan University of Medical S cience in Khorramabad, Iran. almost certainly limits the generalizability of the findings to a broader population. In this study, the relatively short duration between the pre- and post-tests is also a limitation. Long-term follow-up evaluation would be beneficial in assessing the more sustained impact of the educational intervention on self-confidence and satisfaction.

## Conclusion

The results of this study demonstrate the impact of the FCBS training method compared to SBE on self-confidence, satisfaction, knowledge, and learning skill of nursing students. By engaging students in active learning and promoting a learner-centered approach, flipped classroom empower nursing students to acquire and apply clinical skills effectively. It is suggested that incorporating these approaches into nursing curricula can contribute to better preparedness of future nurses, ultimately leading to improved learning outcomes and the delivery of high-quality care. Due to the success of this method in excellent education. Although the complete transfer of practical skills through images and videos is not feasible, this method can be used during pandemics and can reduce the duration or the number of practical training sessions.

## Data Availability

No datasets were generated or analysed during the current study.

## Abbreviations

CPR: Cardio Pulmonary Resuscitation
INACSL: International Nursing Association for Clinical Simulation and Learning
SBE: Simulation Based Education
FCBS: Flipped Classroom-Based Simulation
GPA: Grade-point average
BLS: Basic Life Support
ALS: Advanced Life Support
CVI: Content Validity Index
CVR: Content Validity Ratio
L.M.S: Loestan Medical Science
Clinical trial number: Not applicable
